# Seed bank characteristics in a *Pinus densata* forest and its relationship with vegetation diversity in Southeast Tibet, China

**DOI:** 10.1002/ece3.6603

**Published:** 2020-08-20

**Authors:** Jie Lu, Zhaoqing Li, Tan Gao, Xiaoqin Tang

**Affiliations:** ^1^ Res. Institute of Tibet Plateau Ecology Tibet Agriculture & Animal Husbandry University Nyingchi China; ^2^ Key Laboratory of Forest Ecology in Tibet Plateau Tibet Agriculture & Animal Husbandry University Ministry of Education Nyingchi China; ^3^ Plant Sciences College Tibet Agriculture & Animal Husbandry University Nyingchi China

**Keywords:** aboveground vegetation, *Pinus densata* forest, redundancy analysis, species diversity, underground seed bank

## Abstract

**Objective:**

We aimed to understand the basic characteristics of the underground seed bank of *Pinus densata* forest and its relationship with aboveground vegetation, to provide a theoretical basis for vegetation restoration.

**Methods:**

The study sites were Dongjiu Bridge (DJ), Linji Bridge (LZ), and Birishen Mountain Scenic Spot (RB) in Gongbu Nature Reserve, Southeast Tibet, China. Species composition and distribution pattern of the underground seed bank in *P*. *densata* forest were analyzed. Germination data and field investigations were used to examine the similarities between aboveground vegetation and underground seed banks, and their responses to the environment.

**Results:**

There were 47 species belonging to 27 families in the underground seed bank of the *P. densata* forest. Asteraceae, Rosaceae, Labiatae, and Poaceae were dominant, accounting for 40.4% of the total number of families. Underground seed density was 2,114, 1,952, and 1,141 seeds/m^2^ in DJ, LZ, and BR, respectively. The percentage of different life‐forms in each sampling location was shown to be perennial herbs > shrubs > annual herbs > trees > subshrubs. The Shannon–Wiener diversity index, Margalef richness index, and Simpson's dominance index of each sample showed that species decreased with higher elevation, while the Pielou evenness index showed the opposite trend. Elevation, slope position, slope aspect, and slope were positively correlated with the Pielou evenness index and negatively correlated with the Shannon–Wiener diversity index, Margalef richness index, and Simpson's dominance index. The Sørensen index, comparing the similarity between the underground seed bank and aboveground vegetation of DJ, LZ, and BR was 0.46, 0.35, and 0.31, respectively.

**Conclusion:**

The underground seed bank of *P*. *densata* forest has high seed density and high species richness, but there was little similarity between aboveground vegetation and underground seed bank. Elevation and slope position had a great influence on the uniformity of species distribution.

## Graphical abstract

Compositae, Rosaceae, Labiatae, and Gramineae as dominant species accounted for 40.42% of the total species. Percentage of different life‐forms in each sampling location was shown as perennial herb > shrub > annual herb > tree > subshrub. The elevation and slope position had great influence on the uniformity of species distribution.

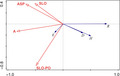

## 1 INTRODUCTION

The underground seed bank is a critical stage in the life history of vegetation, which affects processes such as population size, survival, reproduction, and diffusion, and plays an influential role in maintaining population structure, community stability, and vegetation renewal and restoration (Osuri et al., 2017; Silverman, 2018). The differences in species composition between the vegetation and the seed bank can reflect the succession process (Dölle & Schmidt, 2009). Therefore, the relationship between the seed bank and standing vegetation has been well researched (Amiaud & Touzard, 2004; Takagawa, Washitani, Uesugi, & Tsumura, 2006). The primary source of vegetation renewal is the seed distributed on the surface of the soil, which generally has a strong germination ability, while the deeper seed banks form a durable seed store (Fernández, Vega, & Fontúrbel, 2018).

The number of seeds in the seed bank varies with a change in the composition of aboveground vegetation (Long, 2013). Compared with other ecosystems, the number of seeds in forest soil seed banks is small and is mainly affected by forest age and the succession stage (Perera, 2005). Numerous studies have investigated the species similarity of underground seed banks and aboveground vegetation in tropical and temperate forests, wetlands, and alpine grasslands (Cui et al., 2016; Dalton, Carpenter, Boutin, & Allison, 2017; Douh et al., 2018). However, there are no studies on the relationship between underground seed banks and aboveground vegetation in *P. densata* forest in Southeast Tibet, China. *P*. *densata* is an endemic tree in China and is one of the dominant species in this region. It has an important ecological value in water conservation, soil and water balance, and biodiversity protection (Gao, Mao, & Invarsson, 2012). In recent years, *P*. *densata* has suffered serious degradation from diseases and insect pests, which has seriously affected the stability and sustainability of its population. The underground seed bank is of considerable significance for the renewal and recovery of the species.

In this study, three sampling locations with a typical distribution of *P*. *densata* in the Gongbu Nature Reserve, Southeast Tibet, were used as study sites. Species composition, distribution characteristics, seed bank similarity to aboveground vegetation, and seed bank response to environmental factors in *P*. *densata* forests at the different locations were analyzed.

## 2 MATERIALS AND METHODS

### 2.1 Study environment

**FIGURE 1:**
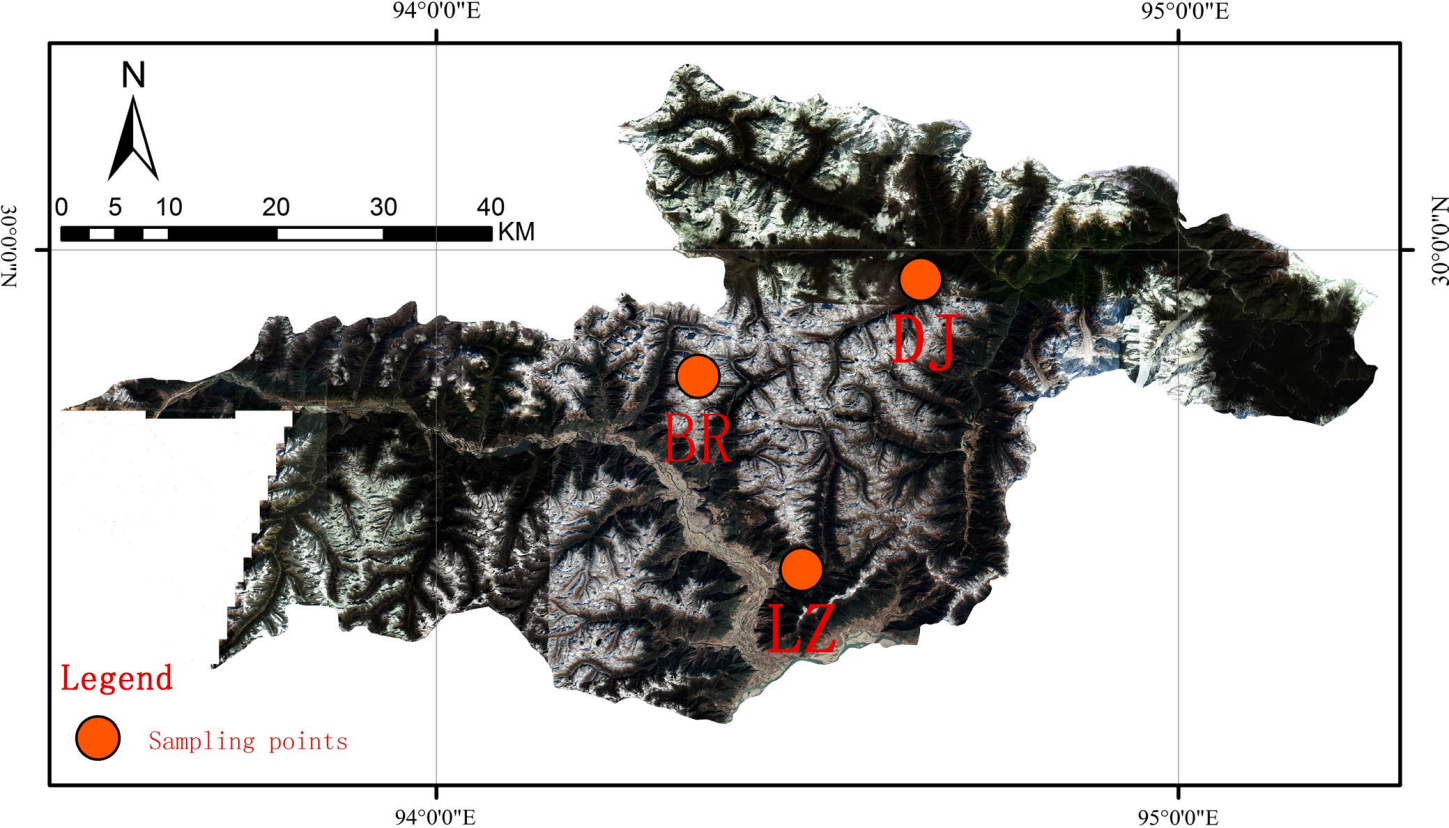
Study area and sampling locations. Red dots represent sampling locations. DJ, LZ, and BR represent Dongjiu Bridge, Linji Bridge, and Birishen Mountain Scenic Spot, respectively. The *x*‐axis represents longitude, and the *y*‐axis represents latitude

The sampling sites (92°09ʹ–98°47ʹE, 26°52ʹ–30°40ʹN) were located in the Gongbu Nature Reserve in Nyingchi City (Figure 1), which is a relatively undisturbed forest area in Southeast Tibet, China (Zhang, 2011). The sites have humid temperate and semi‐humid temperate climate of the mountain region and are affected by the warm and wet monsoon of the Indian Ocean. The average annual temperature is −0.73°C, and the average annual rainfall is 1,134 mm. The average annual relative humidity in this area is 78.8%, and the annual sunshine duration is 1,151 hr (Lu & Fang, 2016).

The Sejila Mountains are the core mountain range in the region. The range shows an east–west trend, a large elevation change, and diverse ecosystems. The dominant plant species from high to low elevation include *Sabina saltuaria*, *Abies georgei* var. *smithii*, *Picea likiangensis* var. *linzhiensis*, *Quercus aquifolioides*, *Sorbus rehderiana*, *Viburnum kansuense*, *Lonicera inconspicua*, *Leptodermis* sp., *Rosa macrophylla* var. *glandulifera*, *Rhododendron nyingchiense*, and *Senecio solidagineus*. The parent material is granite, and the soil type is mountain acid brown soil (Shen, Lu, Hua, & Fang, 2016). The sampling locations were selected in Dongjiu Bridge in Dongjiu Township (DJ), Linji Bridge in Nyingchi Town (LZ), and in Birishen Mountain Scenic Spot in the Bayi District (BR; Figure 1). BR, a scenic area, is affected by a large number of tourists visiting all year round. LZ is close to human habitation and is influenced by human activities to a certain extent, while DJ is far from human habitation.

Slope, slope aspect, and slope position were measured by a compass. Latitude, longitude, and elevation were measured by Global Positioning System (Jiaming, China, GPSMAP631SC).

### 2.2 Seed bank and vegetation survey

The seed bank samples were collected and the numbers of the aboveground plants were measured at DJ, LZ, and BR in March and September 2018, respectively. For trees, three 20 m × 30 m quadrats were set at the bottom, middle, and top of each mountain, respectively, with a total of 27 tree quadrats. We identified the tree species and recorded the height, abundance, diameter at breast height, and crown width of the trees. Based on the five‐point sampling method (Hovind & Rieck, 1961), five 2 m × 2 m shrub quadrats were set at each tree quadrat, and the identify, abundance, height, and coverage of the shrubs in the quadrats were recorded. A total of 135 (45 × 3) shrub quadrats were used. Each shrub quadrat was set a 1 m × 1 m herbaceous quadrat, and data included the identity, abundance, height, and coverage of the herbaceous were also recorded, with a total of 135 herbaceous quadrats (Figure S1). An Apresys laser rangefinder (MINI1800IC) was used to measure the height and coverage of trees and shrubs. For herbaceous, the height was measured using a tape. We also performed a taxonomic identification of the herbaceous in the quadrats.

Soil samples were collected in the same location as the survey of aboveground vegetation. For each site slope, ten 20 cm × 20 cm quadrats were set at the top, middle, and bottom, respectively. For each 20 cm × 20 cm quadrat, soil samples were taken at 0–2 cm and 2–5 cm depths. A total of 180 soil samples were placed in ziplock bags and transferred to the laboratory for the germination experiments. Litter was removed from the ground before soil sampling. Details of sampling locations are shown in Table 1.

**TABLE 1:** Basic characteristics of sampling locations

Zone	LL	*A* (m)	SLO (°)	ASP	DBH (cm)	HT (m)	CW (m^2)^	SA (yr)
DJ	94°49′14.38ʺE 29°57′20.70ʺN	2,400~2,800	33	S	36.26 ± 1.16^a^	18.12 ± 0.45^a^	15.26 ± 1.12^a^	41.17 ± 0.65^a^
LZ	94°30′32.11ʺE 29°34′02.50ʺN	3,100~3,400	32	WS	26.66 ± 2.29^b^	12.61 ± 0.64^b^	23.89 ± 1.75^b^	38.16 ± 0.77^b^
BR	94°22′23.48ʺE 29°34′15.91ʺN	3,000~3,300	22	W	27.25 ± 0.81^b^	12.90 ± 0.22^b^	23.00 ± 1.22^b^	40.15 ± 0.56^a^

Note

DJ, LZ, and BR represent Dongjiu Bridge, Linji Bridge, and Birishen Mountain Scenic Spot, respectively. LL represents longitude and latitude; A represents elevation; SLO represents slope; ASP represents slope aspect; S represents south; WS represents southwest; W represents west; DBH represents diameter at breast height; HT represents height of tree; CW represents crown width; SA represents stand age. Measurement data are represented by mean ± *SD*. Different letters in the same column indicate significant differences (*p* < .05), and the same letters in the same column indicate no significant differences (*p* > .05)

### 2.3 Germination assay

The soils used for the germination experiment were all treated by high‐temperature sterilization at 120°C. The temperature of the germination experiment was controlled to approximately 25°C during the day (12 hr) and approximately 10°C at night (12 hr) in a greenhouse. Seed germination was recorded every 3 days. After planting the seeds, water was added to bring the soil moisture up to 100%, and then, the samples were kept moist by watering based on the soil moisture each day. When seedlings appeared, taxonomic identification was carried out and the species and numbers of seedlings were recorded. If a sample had no new seedlings for 6 consecutive weeks, the germination experiment of the sample was considered to be over. In order to exclude the presence of foreign seeds, three soil samples after high‐temperature treatment were set as blank controls in this experiment. Plant identifications followed the Chinese Virtual Herbarium (http://www.cvh.ac.cn/cnpc; Li, Du, & Guo, 2015). The average density of the seeds in each sample was expressed on a per m^2^ basis.

### 2.4 Statistical analysis

SPSS 17.0 was used to do the data analysis. One‐way analysis of variance (ANOVA) and Duncan's test were used to compare the basic characteristics of three locations, and the species diversity of three locations. Independent‐sample *t* test was carried out for seed bank density analysis of different soil layers. The mean variability was indicated by the standard error.

The Shannon–Wiener diversity index (H), Margalef richness index (R), Simpson's dominance index (D), and Pielou evenness index (E) were used to demonstrate the diversity of seed bank. The Sørensen index (Sc) was used to analyze species similarity between the seed bank and aboveground vegetation. The equations for the indices are as follows:

$$\text{Shannon ‐ Wiener diversity index:}\;H=‐\sum_{i=1}^{S}P_{i}\log_{2}P_{i}$$

$$\text{Margalef richness index}:R=\frac{\left(S‐1\right)}{\ln N}$$

$$\text{Simpson's dominance index}:D=1‐\sum_{i=1}^{S}P_{i}^{2}$$

$$\text{Pielou evenness index}:E=\frac{H}{\ln S}$$

$$\text{Sørensen index}:\text{Sc}=\frac{2j}{a+b}$$

where *P_i_* represents the relative importance value of each species; *N* represents the total number of all types of individual species; *S* represents the number of species in each quadrat; *a* and *b* represent the number of species in the underground seed bank and aboveground vegetation, respectively; *j* represents the number of species shared.

A 9 × 4 environmental factor matrix and a 9 × 4 species diversity matrix were constructed, and Canoco 5.0 was used for redundancy analysis (Braak & Smilauer, 2012).

## 3 RESULTS

### 3.1 Density and species composition in the seed bank

Based on the germination experiment results (Table 2), 47 species of plants belonging to 27 families were identified. Asteraceae (8 species), Rosaceae (5 species), Lamiaceae (3 species), and Poaceae (3 species) accounted for 17.0%, 10.6%, 6.4%, and 6.4% of the total species, respectively. Plant families represented by a single species accounted for 38.3% of the total species. According to the life‐forms of the seed bank, trees, shrubs, and herbaceous plants accounted for 4.3%, 21.3%, and 74.5% of the total species, respectively.

**TABLE 2:** Species composition and density of underground seed banks in *Pinus densata* forests

Family	Species	Life‐form	Seed density (seeds per m^2^)		
			DJ	LZ	BR
Asteraceae	*Artemisia sieversiana*	Perennial herb	25 ± 0	0	0
	*Erigeron multiradiatus*	Perennial herb	63 ± 30	0	33 ± 16
	*Senecio diversifolius*	Perennial herb	25 ± 0	0	25 ± 0
	*Anaphalis spodiophylla*	Perennial herb	88 ± 66	44 ± 26	0
	*Anaphalis margaritacea*	Perennial herb	25 ± 0	0	0
	*Senecio scandens*	Perennial herb	25 ± 0	0	29 ± 14
	*Cirsium lanatum*	Perennial herb	0	0	38 ± 20
	*Youngia stebbinsiana*	Perennial herb	50 ± 31	25 ± 0	0
Rosaceae	*Cotoneaster rubens*	Shrub	25 ± 0	0	0
	*Rosa sericea*	Shrub	0	0	25 ± 0
	*Rubus biflorus*	Shrub	25 ± 0	42 ± 8	56 ± 27
	*Duchesnea indica*	Perennial herb	56 ± 31	5 ± 21	00
	*Fragaria nubicola*	Perennial herb	66 ± 12	48 ± 12	43 ± 4
Lamiaceae	*Micromeria wardii*	Subshrub	44 ± 22	0	25 ± 0.0
	*Elsholtzia densa*	Perennial herb	25 ± 0	0	0
	*Clinopodium chinense*	Perennial herb	102 ± 108	93 ± 44	74 ± 14
Poaceae	*Poa annua*	Perennial herb	75 ± 54	256 ± 196	25 ± 0
	*Festuca ovina*	Perennial herb	35 ± 14	100 ± 97	38 ± 20
	*Deyeuxia scabrescens*	Perennial herb	129 ± 89	65 ± 7	46 ± 28
Leguminosae	*Piptanthus nepalensis*	Shrub	25 ± 0	0	0
	*Desmodium elegans*	Shrub	25 ± 0	0	44 ± 26
Caryophyllaceae	*Stellaria lanata*	Perennial herb	138 ± 65	132 ± 82	81 ± 66
	*Stellaria patens*	Perennial herb	142 ± 88	0	0
Apiaceae	*Bupleurum marginatum*	Perennial herb	0	0	25.0 ± 0.0
	*Hydrocotyle sibthorpioides*	Perennial herb	42 ± 24	38 ± 14	0
Rubiaceae	*Leptodermis potanini*	Shrub	25 ± 0	0	25 ± 0
	*Galium aparine* var. *tenerum*	Annual herb	119 ± 67	0	0
Scrophulariaceae	*Hemiphragma heterophyllum*	Perennial herb	0	33 ± 12	25 ± 0
	*Verbascum thapsus*	Perennial herb	0	0.0	50 ± 24
Pinaceae	*Pinus densata*	Tree	51 ± 5	51 ± 6	169 ± 15
Aceraceae	*Acer tetramerum*	Tree	38 ± 20	0.0	0
Buddlejaceae	*Buddleja crispa*	Shrub	25 ± 0	25 ± 0	0
Rhamnaceae	*Berchemia yunnanensis*	Shrub	0	0	75 ± 35
Guttiferae	*Hypericum hookerianum*	Shrub	105 ± 53	163 ± 43	25 ± 0
Plantaginaceae	*Plantago asiatica*	Perennial herb	50 ± 0	238 ± 112	0
Dipsacaceae	*Triplostegia glandulifera*	Perennial herb	75 ± 35	25 ± 0	0
Euphorbiaceae	*Euphorbia wallichii*	Perennial herb	0	75 ± 54	0
Violaceae	*Viola szetschwanensis*	Perennial herb	28 ± 14	50 ± 0	0
Campanulaceae	*Campanula colorata*	Perennial herb	66 ± 36	93 ± 51	0
Onagraceae	*Circaea alpina*	Perennial herb	44 ± 22	0	0
Boraginaceae	*Hackelia brachytubum*	Perennial herb	0	0	25 ± 0
Oxalidaceae	*Oxalis corniculata*	Perennial herb	35 ± 17	100 ± 0	0
Geraniaceae	*Geranium sibiricum*	Perennial herb	36 ± 18	68 ± 18	64 ± 17
Polygonaceae	*Polygonum runcinatum*	Annual herb	73 ± 59	76 ± 62	25 ± 0
Chenopodiaceae	*Acroglochin persicarioides*	Annual herb	25 ± 0	0	0
Solanaceae	*Solanum nigrum*	Annual herb	25 ± 0	0	0
Cyperaceae	*Bulbostylis densa*	Annual herb	40 ± 23	63 ± 27	50 ± 0
Number of species			39	24	25
Total density			2,114 ± 123	1,952 ± 147	1,141 ± 42

Note

Mean ± *SE*.

A total of 39 species belong to 23 families were identified in the seed bank in DJ, and the seed density was 2,114 ± 123 seeds/m^2^. There were 24 species belong to 20 families in LZ, and the seed bank density was 1,952 ± 147 seeds/m^2^. In BR, the seed bank density was 1,141 ± 42 seeds/m^2^, and these seeds belong to 16 genera out of 25 families. Herbaceous plants dominated the seed bank, followed by shrubs. Trees formed the smallest proportion. The dominance of perennial herbs can be attributed to their many advantages such as a wide diversity, wide distribution, and easily achieved germination conditions.

### 3.2 Life‐form of the species in the seed bank

**FIGURE 2:**
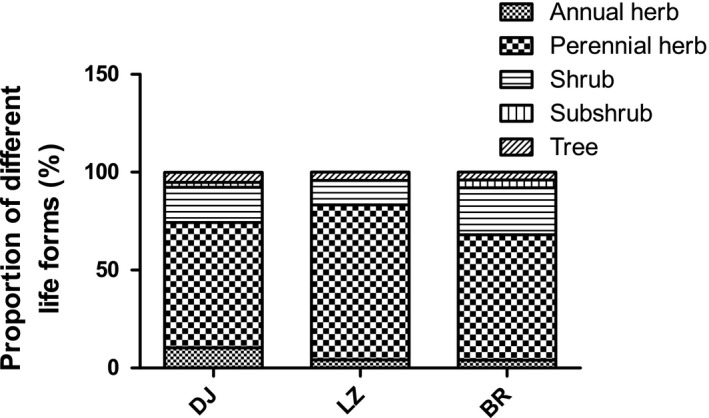
Life‐forms of the standing vegetation in each sampling location. DJ, LZ, and BR represent Dongjiu Bridge, Linji Bridge, and Birishen Mountain Scenic Spot, respectively

Plants with different life‐forms were found in different sampling locations. Figure 2 shows that the seed banks in *P. densata* at DJ and BR were composed of annual herbs, perennial herbs, shrubs, subshrubs, and trees, but there were no subshrubs in the underground seed banks of LZ. The percentage of different life‐forms in each sampling location was shown to be perennial herbs > shrubs>annual herbs > trees>subshrubs. Perennial herbs accounted for at least 64% of life‐forms in all three sampling locations. The proportion of trees in the three sampling sites was <6%.

### 3.3 Vertical distribution pattern of the seed bank

**FIGURE 3:**
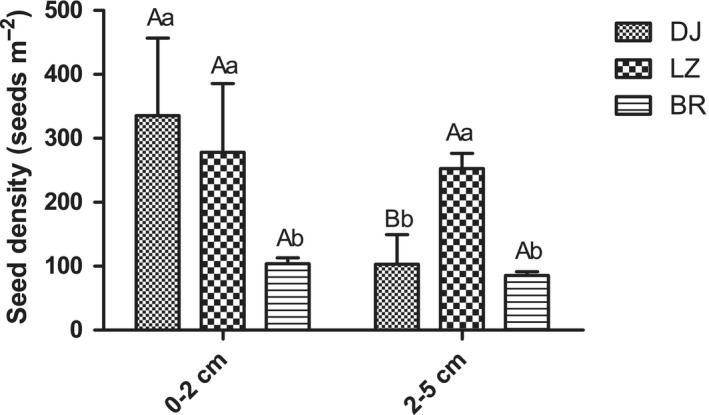
Distribution characteristics of the underground seed bank at different depths. Different lowercase letters indicate significant differences between different points in the same soil layer, and different uppercase letters indicate significant differences between different soil layers at the same sampling location

In this study, the seed bank density in the three sampling locations showed a trend of lower seed density in deeper soils (Figure 3). In the 0–2 cm group, the seed density in BR was less than that of DJ and LZ (*p* < .05), while no significant difference in underground seed bank density between DJ and LZ was observed (*p* > .05). In the 2–5 cm group, the seed bank density of *P. densata* forest at LZ varied significantly from the seed bank densities at DJ and BR (*p* < .05). In both depth layers, the seed bank of BR had the lowest density.

### 3.4 Species diversity of the underground seed bank

The Shannon–Wiener diversity index and Margalef index in the three sites showed the trend of DJ > LZ > BR, but there was no significant difference (*p* > .05). There was also no significant difference among the three sites in Simpson's dominance index and Pielou evenness index (*p* > .05; Table 3).

**TABLE 3:** Species diversity in underground seed banks of *Pinus densata* forests in different sampling locations

Zone	Shannon–Wiener	Margalef	Simpson	Pielou
DJ	1.11 ± 0.09a	4.30 ± 0.69a	0.89 ± 0.02a	0.35 ± 0.01a
LZ	1.01 ± 0.10a	3.16 ± 0.37a	0.86 ± 0.04a	0.35 ± 0.03a
BR	0.97 ± 0.08a	3.05 ± 0.52a	0.86 ± 0.02a	0.37 ± 0.00a

Note

DJ, LZ, and BR represent Dongjiu Bridge, Linji Bridge, and Birishen Mountain Scenic Spot, respectively. Shannon–Wiener represents Shannon–Wiener diversity index; Margalef represents Margalef richness index; Simpson represents Simpson's dominance index; Pielou represents Pielou evenness index. Different letters in the same column indicate significant differences (*p* < .05), and the same letters in the same column indicate no significant differences (*p* > .05). Measurement data are represented by mean ± *SE*.

### 3.5 Similarity analysis between the underground seed bank and vegetation

**FIGURE 4:**
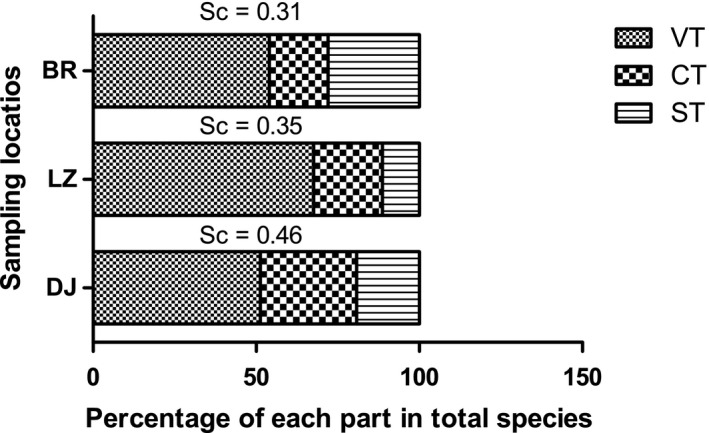
The similarity between underground seed banks and aboveground vegetation in different sampling locations. DJ, LZ, and BR represent Dongjiu Bridge, Linji Bridge, and Birishen Mountain Scenic Spot, respectively. Se represents the Sørensen index. VT represents the percentage of aboveground vegetation in total species. CT represents the percentage of common species in total species. ST represents the percentage of underground seed bank in total species

The results of the plot survey (Table 4) indicated that DJ (63 species) and LZ (63 species) had more aboveground vegetation species than BR (36 species), and DJ (38 species) had the largest number of seed species. The shared species between the seed bank and vegetation in DJ, LZ, and BR decreased in order, which were 23, 15, and 9, respectively. The percentage of shared species in the seed bank was higher than the percentage of shared species in vegetation in each sampling location. The number of shared species accounted for more than 60% of the seed bank in both DJ and LZ. The Sørensen index of DJ, LZ, and BR was 0.46, 0.35, and 0.31, respectively (Figure 4).

**TABLE 4:** Population structure of underground seed bank and aboveground vegetation in *Pinus densata* forest

Item	DJ	LZ	BR
Number of aboveground vegetation species	63	63	36
Number of underground seed bank species	38	23	23
Number of species shared	23	15	9
Percentage of common species in aboveground vegetation (%)	36.5	23.8	25.0
Percentage of common species in the underground seed bank (%)	60.5	65.2	39.1

Note

DJ, LZ, and BR represent Dongjiu Bridge, Linji Bridge, and Birishen Mountain Scenic Spot, respectively.

### 3.6 Redundancy analysis of seed bank

**FIGURE 5:**
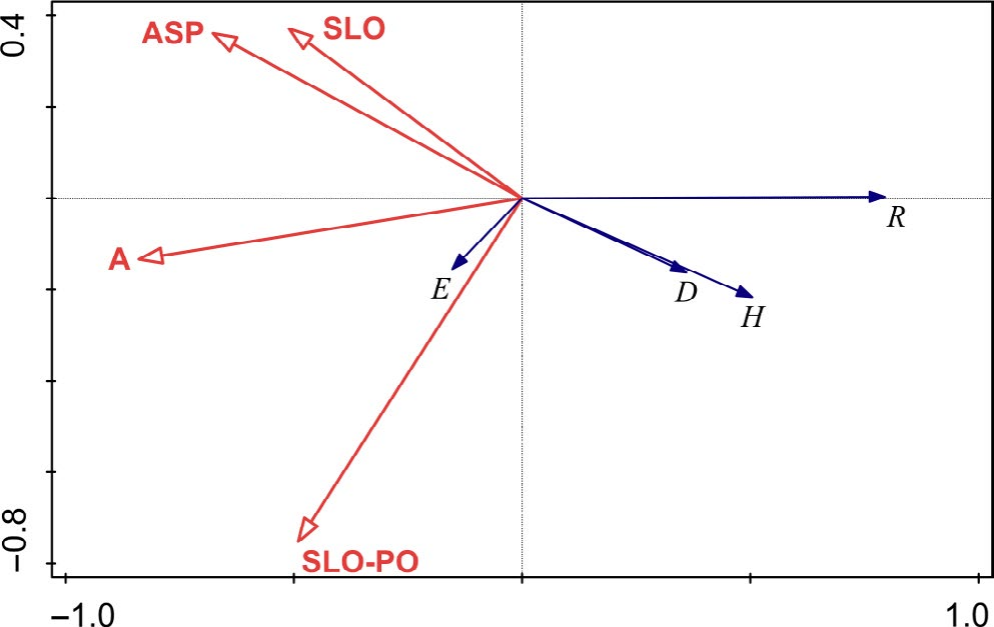
Redundancy analysis between species diversity and environmental factors. A represents elevation; SLO represents slope; ASP represents slope aspect; SLO‐PO represents slope position. H represents Shannon–Wiener diversity index; R represents Margalef richness index; D represents Simpson's dominance index; E represents Pielou evenness index

The redundancy analysis of the seed bank and environmental factors in *P. densata* forest showed that environmental factors such as elevation, slope position, slope aspect, and slope had a great influence on the diversity characteristics of seed bank groups in the forest (Figure 5). Furthermore, the first RDA (Axis I) axis explained 62.2% of the variability in the data, while Axis II explained 0.1%. Elevation, slope position, slope aspect, and slope were positively related to the Pielou evenness index and negatively related to the Shannon–Wiener diversity index, Margalef index, and Simpson's dominance index.

## 4 DISCUSSION

The underground seed bank plays a significant role in vegetation regeneration. The composition and size of the seed bank have been the main research focus (Yang, 2001; Yuan, Liu, Li, & Li, 2007). Some investigations have suggested that the characteristics of the seed bank reflect the composition of surface vegetation (Hirayama, Yamada, Inui, & Takahashi, 2019; Sousa et al., 2017). However, other studies have found that the composition of the seed bank bears little resemblance to the surface vegetation (Dölle & Schmidt, 2009; Savadogo, Sanou, Dayamba, Bognounou, & Thiombiano, 2016). Bakker et al. showed that only 24% of species in the surface vegetation were found in the seed bank (Bakker, de Graaf, Ernst, & van Bodegom, 2005). Research has shown that the similarity between forest seed banks and plants is <60%, and most are distributed between 20% and 40% (Hopfensperger, 2007). The results of the present study indicated that the similarity between the aboveground vegetation and the underground seed bank of *P. densata* forest was low. There may be several reasons for this: (a) germination experiments may not completely meet the germination conditions for some plants, which would lead to a failure of germination and, thus, reduce the number of species in a seed bank. For example, seeds of honeysuckle (*Lonicera maackii*) need to be chilled to break dormancy and allow germination (McEwan, Arthur‐Paratley, Rieske, & Arthur, 2010). (b) *P. densata* forests are in the late stable stage of community succession, and some studies have shown that, with an increase in succession age, the common species existing in terrestrial vegetation and seed bank decrease (Bistea & Mahy, 2005; Vilà & Gimeno, 2007). (c) Some seeds that mature and fall to the surface only stay on the surface for a short time, and either take root or die quickly; these seeds contribute little to the underground seed bank. In addition, threats such as pests and diseases, animal transport and feeding, and seed mildew may affect the composition of seed bank species (Kaewnango & Prasertsak, 2018; Pu & Zhu, 2017).

Research has found that when the underground seed bank is analyzed vertically, the number of seeds in the soil near the surface is higher, and the seed number gradually decreases with increasing depth (Leck & Simpson, 1995), which is consistent with the results of this study in DJ. Generally, the seeds fall to the topsoil first and then enter the deeper soil through processes such as animal trampling or disturbance. Therefore, most of the seeds that fall from plants stay in the surface soil rather than deeper layers. Only a few seeds will be driven into the deep soil by external forces. There are various other factors that may change the seed distribution pattern, such as the human activities, ability of the seed to spread, and changes in the environment (O'Donnell, Fryirs, & Leishman, 2014).

The seed bank density is substantially the basis of natural vegetation restoration (Brown & Venable, 1986; Klaus et al., 2018). The seed density of DJ, LZ, and BR were 2,114 seeds/m^2^, 1,952 seeds/m^2^, and 1,141 seeds/m^2^, respectively. The discrepancies in seed bank density among diverse forest types may be related to regional environment, climate type, vegetation characteristics, terrain characteristics, and elevation factors (Graham & Page, 2018; dos Santos, da Silva, dos Santos, & de Lima Araújo, 2018; Tiebel, Huth, & Wagner, 2018). For example, Douh et al. explored the features of seed banks in two types of forest soils, clay and sand, and found that their seed densities were 330 seeds/m^2^ and 247 seeds/m^2^ (Douh et al., 2018). Seed density in the rainy (1,649 seeds/m^2^) and dry (854 seeds/m^2^) seasons of the same forest also varied greatly (dos Santos, Fraga, da Silva, de Araújo, & de Lima Araújo, 2016).

In this study, the Shannon–Wiener diversity index, Margalef index, and Simpson's dominance index at all points showed the trend DJ > LZ>BR, while the Pielou evenness index showed the opposite trend. The results of redundancy analysis showed that elevation, slope position, slope aspect, and slope were positively correlated with the Pielou evenness index and negatively correlated with the Shannon–Wiener diversity index, Margalef index, and Simpson's dominance index. Elevation and slope position had a greater influence on species distribution uniformity. Under certain climatic conditions, topographic factors form a variety of ecological environments through spatial redistribution of ecological factors such as light, temperature, and water, thereby indirectly affecting the growth of plants (Li, 2013). Elevation is the main factor affecting the distribution of water and heat conditions in mountain areas, which affects the vertical distribution of plants and the diversity of plants (Li, 2017). The slope affects the direction of water and soil nutrients in the vertical direction, which in turn affects the distribution of plants (Jiang, 2014). However, there was a considerable contrast in seed density between LZ and BR, and the elevation and slope aspect of the two sites were similar. This result may be due to the fact that BR is greater disturbed by human activities, which probably reduce seed productivity.

## CONFLICT OF INTERESTS

The authors declare that they have no competing interests.

## AUTHOR CONTRIBUTION

**Jie Lu:** Conceptualization (equal); Formal analysis (equal); Methodology (equal); Writing‐review & editing (equal). **Zhaoqing Li:** Conceptualization (equal); Data curation (equal); Formal analysis (equal); Methodology (equal); Writing‐original draft (equal). **Tan Gao:** Data curation (equal); Formal analysis (equal); Methodology (equal); Writing‐review & editing (equal). **Xiaoqin Tang:** Conceptualization (equal); Formal analysis (equal).

## Supporting information

Fig S1

## Data Availability

The datasets used and analyzed during the current study are available from the corresponding author on reasonable request.
