# Supplementary figures and images for: Seed bank characteristics in a Pinus densata forest and its relationship with vegetation diversity in Southeast Tibet, China
Source: Ecol Evol. 2020 Aug 20;10(17):9214–22. doi: 10.1002/ece3.6603 (PMC7487222; doi:10.1002/ece3.6603)

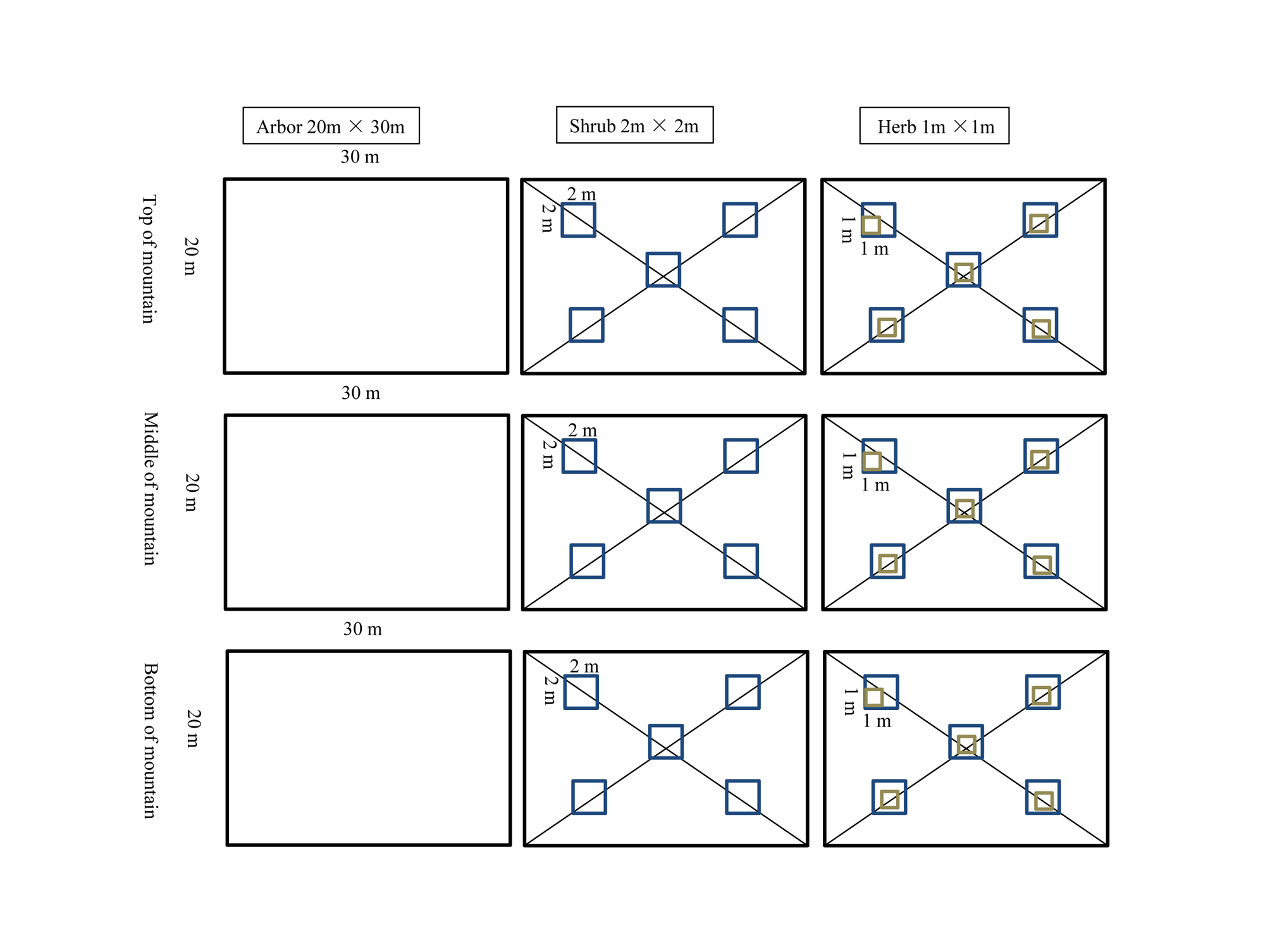

Supplement: Supplementary file 1 — Fig S1 [file ECE3-10-9214-s001.tif]
